# Thermocoagulation versus cryotherapy for the treatment of cervical precancers

**DOI:** 10.1111/jog.14520

**Published:** 2020-10-21

**Authors:** Lyufang Duan, Hui Du, Jerome L. Belinson, Zhihong Liu, Aimin Xiao, Shuangyan Liu, Liwei Zhao, Chun Wang, Xinfeng Qu, Ruifang Wu

**Affiliations:** ^1^ Department of Gynecology and Obstetrics Peking University Shenzhen Hospital Shenzhen China; ^2^ Shenzhen Key Laboratory on Technology for Early Diagnosis of Major Gynecologic Diseases Shenzhen China; ^3^ Preventive Oncology International Cleveland Heights Ohio USA; ^4^ Women's Health Institute, Cleveland Clinic Cleveland Ohio USA; ^5^ Sanming Project of Medicine in Shenzhen Peking University Shenzhen Hospital Shenzhen China

**Keywords:** cervical intraepithelial neoplasia, complication, cryotherapy, efficacy, thermocoagulation, treatment

## Abstract

**Aim:**

To compare thermocoagulation and cryotherapy for treatment of high‐grade cervical intraepithelial neoplasia (CIN).

**Methods:**

From May 2017 to May 2018, women with CIN2/3 were randomized to thermocoagulation or cryotherapy at Peking University Shenzhen Hospital. Follow‐up at 4 and 8 months included cytology and human papillomavirus (HPV) testing. Women who were HPV‐positive or had atypical squamous cells of undetermined significance or higher‐grade disease underwent colposcopy/biopsy.

**Results:**

Among 149 women enrolled, 74 were randomized to thermocoagulation, and 75 to cryotherapy (excluded four were immediately referred for thermocoagulation due to large lesions). At follow‐up, there was no difference between the thermocoagulation and cryotherapy groups in HPV‐negative (4/8 months: 72.5%/86.2% vs 68.6%/80.6%) and pathology‐negative (97.1%/98.5% vs 94.3%/92.3%) rates (all *P* > 0.05). The cytology‐negative rate was similar for thermocoagulation and cryotherapy at 4 months (79.7% vs 78.9%, *P* > 0.05), but higher for thermocoagulation at 8 months (100% vs 88.7%, *P* < 0.05). No lesions were observed among the four referral women at follow‐up. As compared with cryotherapy, thermocoagulation was associated with shorter duration of treatment and less vaginal discharge, but higher pain during application and longer bleeding after treatment.

**Conclusion:**

Thermocoagulation was as effective and safe as cryotherapy and might be easily applied to treat high‐grade cervical lesions.

## Introduction

Cervical intraepithelial neoplasia grade 2 (CIN2) and CIN3 are considered precancerous lesions that may develop into cervical cancer.^1^ Therefore, it is very important to treat high‐grade CIN. The guidelines of the American College of Obstetricians and Gynecologists recommend that, with the exception of pregnant women, CIN2 or CIN3 confirmed by colposcopy‐directed biopsy should be treated in the form of either ablation or excision to prevent progression to cervical cancer.^2^

Currently, cryotherapy is the ablation modality that is most used to treat ectocervical premalignant cervical lesions. Disease‐free rates after treatment with cryotherapy are reported as 92% for CIN2 and 85% for CIN3^3^; these rates are generally comparable to those of excisional procedures such as loop electrosurgical excision procedure (LEEP),^4^ and cryotherapy does not affect subsequent pregnancy rates.^5^ For low‐ and middle‐income countries, the World Health Organization recommends that cryotherapy should be used to treat women who screen positive for CIN after primary human papillomavirus (HPV) testing or visual inspection with acetic acid, even without colposcopy‐ or histology‐verified disease.^6^ This ‘single visit approach’ is likely to significantly increase the demand for cryotherapy in these countries; however, there are problematic issues such as the cost, availability and transport of the large gas tanks.

A more portable technology would have significant advantages for the treatment of cervical precancers, but it is important to maintain the many benefits of cryotherapy. To fill this role, the technology of ‘cold coagulation’, more recently referred to as ‘thermocoagulation’, may be a strong candidate. This technique was originally introduced in 1965 by Kurt Semm.^7^ Studies using older electric devices have demonstrated that thermocoagulation is efficacious and performs similarly to cryotherapy^8^, ^9^, ^10^, ^11^; however, most of those studies were retrospective. In addition, they were based on older electric devices, which were restricted by needing an electricity supply.

The new Liger thermal coagulator (Liger HTU‐100, Liger Medical) is portable with a rechargeable lithium ion battery in the handle to provide the electrical energy for treatment. It is reported to reach the treatment temperature in 6 s and to provide approximately 30 treatments per battery charge depending on treatment parameters. Based on the size of the transformation zone (the area where precancerous changes occur on the cervix), more than one application may be necessary to cover the whole area at risk.

The aims of this study were to determine the effectiveness of thermocoagulation as compared with cryotherapy for the treatment of CIN2‐3.

## Methods

### Study design and sample

The present prospective randomized trial was conducted in the Center for Cervical Diagnosis, Peking University Shenzhen Hospital (PUSH), Shenzhen, China, among women from Shenzhen referred to the clinic from PUSH and other facilities between May 10, 2017, and May 28, 2018. The study was approved by the ethics committee of PUSH (IRB code PUSH2016[37]). All participants consented to being randomized to treatment with either thermocoagulation or cryotherapy.

All participants were referred to the study clinic owing to abnormal liquid‐based cytology (atypical squamous cells of undetermined significance [ASCUS] or higher‐grade cytology) or a positive test for high‐risk HPV genotypes, and had colposcopy performed by a staff gynecologic colposcopy specialist. The study inclusion criteria were nonpregnancy, age 20–49 years, satisfactory colposcopy, histologic confirmation of CIN2/3, negative endocervical curettage and no vaginal lesions. Women were excluded if they had a history of invasive cervical treatment or had been previously vaccinated for HPV. Cases randomly assigned to the cryotherapy group could be referred for thermocoagulation if the lesion was too large that could not be covered by the cryo‐probe.

All participants signed approved informed consent, and were randomized to thermocoagulation or cryotherapy treatment by the lottery method. Sociodemographic and clinical characteristics were recorded for all women.

### Treatment procedure

A full explanation of the study was given to all women before treatment. The participants were informed that menstruation‐like cramps might be experienced during therapy, and that watery vaginal discharge or bleeding may occur after treatment. The women were given a diary card and detailed information on how to complete it.

The treatment was performed under colposcopic guidance without anesthesia. For thermocoagulation, the Liger device with a 19‐mm probe and 3‐mm nipple was placed on the cervix at room temperature and then activated. It took 6 s to reach temperature; the treatment time was then 45 s at 100°C as per the manufacturer's instructions. On completion of the procedure, the probe was withdrawn. If the size or shape of the transformation zone required a second or third application for adequate coverage, a flat 19‐mm probe was used with the same treatment parameters.

For cryotherapy, a MedGyn cryotherapy (MedGyn) device with a 19‐mm probe and 3‐mm nipple was placed on the cervix at room temperature and then activated. The treatment was 3 min of freezing, followed by 5 min of thawing and then 3 min of freezing using CO_2_. Time was monitored with a stopwatch. The minimum freezing range was more than 2 mm beyond the edge of the target lesion. At the end of treatment, the probe was removed from the frozen tissue in accordance with standard cryotherapy procedures.

### Follow‐up examination

Follow‐up visits were conducted at 4 and 8 months. Cytology and Cobas 4800 HPV testing (Roche Molecular) was performed at each, and data were recorded from the participant's diary card at 4 months. Colposcopy and/or biopsy was carried out for all women who tested positive for HPV and/or had cytology of ASCUS or higher‐grade.

A protocol of directed and random biopsies was used in the study. Both colposcopists performed colposcopy and pathologists made histological diagnosis blinding to the interventions. If a participant tested negative for HPV and cytology, and did not have a CIN2+ lesion by colposcopy‐directed cervical biopsy, the case was recorded as a clinical cure. If a participant was diagnosed with CIN2/3 at 4 or 8 months, she was referred for LEEP in the case of CIN3, and either continued with follow‐up or was treated with LEEP (depending on lesion size, age, and pregnancy desires) in the case of CIN2.

### Data analysis

In the randomized controlled trial, the sample sizes of the noninferiority test for two proportions were calculated, namely, the significance level *a* = 0.025, the power (1 − β) = 0.8, high‐grade squamous intraepithelial lesion (HSIL) cure rate of cryotherapy and thermocoagulation were both 92%. The sample allocation ratio of the two treatments was 1:1, noninferiority difference was −0.13. The PASS software calculated that the total sample size was 138, with cryotherapy 69 and thermocoagulation 69. Taking into account the problem of loss of follow‐up patients, the sample size was increased to 149 cases. The software platform spss version 22.0 (IBM) was used for all analyses. Quantitative data were expressed as mean ± standard deviation or median (range). Data that conformed to a normal distribution were analyzed by *t*‐test; data with a non‐normal distribution were analyzed by a nonparametric rank sum test. Qualitative data were analyzed by χ^2^ test or Fisher exact test. All tests were performed at a significance level of 0.05.

## Results

### Clinical characteristics of the study women

During the study period, 149 women met the inclusion criteria and were enrolled in the study. Of these, 74 were randomized to thermocoagulation and 75 to cryotherapy. In the cryotherapy group, four women (CIN2, *n* = 1; CIN3, *n* = 3) were referred immediately for thermocoagulation owing to large lesions that could not be covered by the cryo‐probe.

Among the remaining 145 women, there were no differences in age, body mass index (calculated as weight in kilograms divided by the square of height in meters), parity, pretreatment cytology, HPV, pathologic results or type of transformation zone between the thermocoagulation group and the cryotherapy group (*P* > 0.05) (Table 1). The women who had their HIV status checked were all negative.

**Table 1:** Clinical characteristics of the study women by treatment group^†^

Characteristic	Thermocoagulation (*n* = 74)	Cryotherapy (*n* = 71)	*P*‐value
Age (years)	31.5 ± 5.2	31.2 ± 5.8	0.738
BMI	20.37 ± 2.80	20.43 ± 2.61	0.907
Pregnancy ≥1	56 (75.7)	53 (74.6)	0.886
Nulliparous	32 (43.2)	33 (46.5)	0.695
Baseline cytology			
NILM	20 (27.0)	19 (26.8)	0.218
ASCUS	11 (14.9)	19 (26.8)	
LSIL	21 (28.4)	20 (28.2)	
HSIL/ASC‐H	22 (29.7)	13 (18.3)	
Baseline HPV			
HPV16	28 (37.8)	23 (32.4)	0.692
HPV18	2 (2.7)	2 (2.8)	
Non‐HPV16/18	52 (70.3)	52 (73.2)	
Pathology			
CIN2	55 (74.3)	60 (84.5)	0.130
CIN3	19 (25.7)	11 (15.5)	
TZ			
Type I	57 (77.0)	47 (66.2)	0.148
Type II	17 (23.0)	24 (33.8)	

^†^ Values are given as mean ± standard deviation or number (%).

ASC‐H, atypical squamous cells, cannot exclude HSIL; ASCUS, atypical squamous cells of undetermined significance; BMI, body mass index (calculated as weight in kilograms divided by the square of height in meters); CIN, cervical intraepithelial neoplasm; HPV, human papillomavirus; HSIL, high‐grade squamous intraepithelial lesion; LSIL, low‐grade squamous intraepithelial lesion; NILM, negative for squamous intraepithelial lesion or malignancy; TZ, transformation zone.

### Follow‐up data and complications of therapy

**Figure 1:**
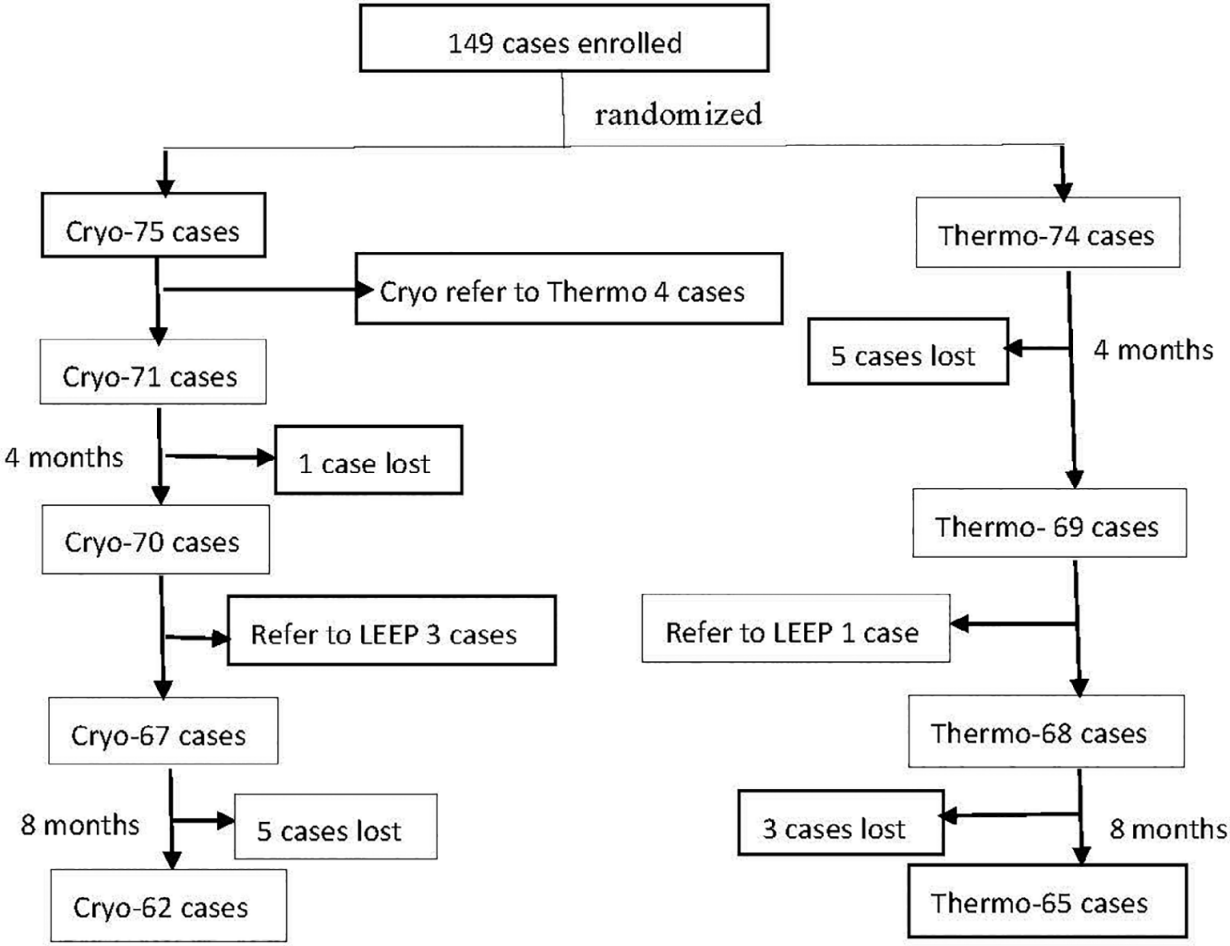
Flowchart showing treatment and follow‐up of the study women.

Figure 1 shows a flowchart of the treatment and follow‐up of participants. Six women (thermocoagulation group, *n* = 5; cryotherapy group, *n* = 1) were lost to follow up at 4 months after treatment. The results of cytology, HPV genotyping and pathology of 143 women (including the four immediately referred to thermocoagulation) at 4 months after treatment are shown in Table 2. Of these, four women subsequently underwent LEEP for CIN2 and CIN3 (thermocoagulation group, *n* = 1; cryotherapy group, *n* = 3) and were not included further in the analysis.

**Table 2:** Clinical data of the study women at 4 months after treatment

Group	*n*	Cytology				HPV				Pathology			
		NILM	ASCUS	LSIL	HSIL/ASC‐H	−	16+	18+	Non 16/18+	−	CIN1	CIN2	CIN3
Thermo^†^	69	55	8	4	2	50	3	1	18	21	2	1	1
Cryo^†^	70	55	7	8	0	48	8	0	16	22	2	2	2
Refer T^‡^	4	4	0	0	0	4	0	0	0				

^†^ Six women were lost to follow up at 4 months after treatment (thermocoagulation, *n* = 5; cryotherapy, *n* = 1). In thermocoagulation group, two patients had double infections of HPV16 and non‐HPV16/18, one patient had double infections of HPV18 and non‐HPV16/18 after treatment. In cryotherapy group, two patients had double infections of HPV16 and non‐HPV16/18 after treatment.

^‡^ Four women randomized to the cryotherapy group who were immediately referred for thermocoagulation due to large lesions uncoverable by the cryoprobe.

ASC‐H, atypical squamous cells, cannot exclude HSIL; ASCUS, atypical squamous cells of undetermined significance; CIN, cervical intraepithelial neoplasm; Cryo, cryotherapy; HPV, human papillomavirus; HSIL, high‐grade squamous intraepithelial lesion; LSIL, low‐grade squamous intraepithelial lesion; NILM, negative for squamous intraepithelial lesion or malignancy; Thermo, thermocoagulation.

At 8 months, a further eight women (thermocoagulation group, *n* = 3; cryotherapy group, *n* = 5) were lost to follow up. The results of cytology, HPV genotyping and pathology of 131 women (including the four referral women) at 8 months after treatment are shown in Table 3.

**Table 3:** Clinical data of the study women at 8 months after treatment

Group	*n*	Cytology			HPV					Pathology			
		NILM	ASCUS	LSIL	HSIL/ASC‐H	−	16+	18+	Non 16/18+	−	CIN1	CIN2	CIN3
Thermo^†^	65	65	0	0	0	56	1	1	7	6	3	0	0
Cryo^†^	62	55	5	0	2	50	2	0	10	11	1	1	1
Refer T^‡^	4	4	0	0	0	4	0	0	0				

ASC‐H, atypical squamous cells, cannot exclude HSIL; ASCUS, atypical squamous cells of undetermined significance; CIN, cervical intraepithelial neoplasm; Cryo, cryotherapy; HPV, human papillomavirus; HSIL, high‐grade squamous intraepithelial lesion; LSIL, low‐grade squamous intraepithelial lesion; NILM, negative for squamous intraepithelial lesion or malignancy; Thermo, thermocoagulation.

^†^ A further eight women were lost to follow up at 8 months after treatment (thermocoagulation, *n* = 3; cryotherapy, *n* = 5).

^‡^ Four women randomized to the cryotherapy group who were immediately referred for thermocoagulation due to large lesions uncoverable by the cryoprobe.

There were no differences at the 4‐/8‐month follow‐up between the thermocoagulation and cryotherapy groups in the HPV‐negative rate (72.5%/86.2% vs 68.6%/80.6%, both *P* > 0.05) and pathology‐negative rate (97.1%/98.5% vs 94.3%/92.3%, both *P* > 0.05) (Table 4). The cytology‐negative rate of the thermocoagulation group was the same as that of the cryotherapy group at the 4‐month follow‐up (79.7% vs 78.9%, *P* > 0.05), but higher than that of the cryotherapy group at 8 months (100% vs 88.7%, *P* < 0.05). The four women who were initially referred from cryotherapy to thermocoagulation due to lesion size, each underwent four applications of thermocoagulation, and no lesions were observed at either (4/8 months) follow‐up visit. In the thermocoagulation group, the thermocoagulation applications per treatment were determined in accordance with the size of the lesion. The number of women who underwent 1, 2, 3 and 4 applications of the thermoprobe was 30, 25, 14 and 5, respectively (Table 5). There was no difference in cytology‐negative rate, HPV‐negative rate or clinical cure rate by the number of treatment applications at either the 4‐ or 8‐month follow‐up (both *P* > 0.05).

**Table 4:** Cytology and HPV‐negative rate and clinical cure rate after treatment^†^

Group	Cytology negative		HPV negative		Clinical cure	
	4 months	8 months	4 months	8 months	4 months	8 months
Thermo	55 (79.7)	65 (100)	50 (72.5)	56 (86.2)	67 (97.1)	65 (98.5)
Cryo	55 (78.6)	55 (88.7)	48 (68.6)	50 (80.6)	66 (94.3)	60 (92.3)
Refer T^‡^	4 (100)	4 (100)	4 (100)	4 (100)	4 (100)	4 (100)
*P* value^§^	0.738	0.006	0.475	0.264	0.375	0.078

^†^ Values are given as number (percentage).

^‡^ Four women randomized to the cryotherapy group who were immediately referred for thermocoagulation due to large lesions uncoverable by the cryoprobe.

^§^ Thermocoagulation versus cryotherapy.

Cryo, cryotherapy; HPV, human papillomavirus; Thermo, thermocoagulation.

**Table 5:** Relationship between treatment applications of thermocoagulation and outcome

Treatment applications	Case (*n*)	4 months				8 months			
		Case	Cytology negative, *n* (%)	HPV negative, *n* (%)	Cure rate, *n* (%)	Case	Cytology negative, *n* (%)	HPV negative, *n* (%)	Cure rate, *n* (%)
1	30	28	21 (75.0)	20 (71.4)	26 (92.9)	26	26 (100)	20 (76.9)	25 (96.3)
2	25	22	19 (86.4)	17 (77.3)	22 (100)	21	21 (100)	20 (95.2)	21 (100)
3	14	14	10 (71.4)	11 (78.6)	14 (100)	13	13 (100)	13 (100)	13 (100)
4	5	5	5 (100)	2 (40)	5 (100)	5	5 (100)	3 (60)	5 (100)
*P*‐value			0.415	0.366	0.389		1.000	0.051	0.677

The duration of thermocoagulation was shorter than that of cryotherapy (92.3 ± 49.1 vs 660 ± 0.0 s, *P* < 0.001), but quantify pain using visual analogue scale in cryotherapy was lower than that in thermocoagulation during treatment (2.2 ± 1.3 vs 3.0 ± 2.4, *P* < 0.05). No vaginal bleeding occurred during treatment in either group. As compared with cryotherapy, thermocoagulation led to a shorter duration and smaller quantity of post‐therapy vaginal discharge, but a longer duration of vaginal bleeding after treatment (Table 6).

**Table 6:** Vaginal discharge and vaginal bleeding after treatment^†^

Group	Vaginal discharge			Vaginal bleeding		
	No. of women (%)	Start time, days	Duration, days	No. of women (%)	Start time, days	Duration, days
Thermo	69 (100)	1	17.2 ± 6.9	54 (78.3%)	10	10.6 ± 5.8
Cryo	70 (100)	0	20.8 ± 6.5	17 (24.3%)	7	5.6 ± 3.2
*P*‐value	1.000	0.029	0.002	<0.001	0.149	0.001

^†^ Values are given as mean ± standard deviation or median, number (%).

Cryo, cryotherapy; Thermo, thermocoagulation.

No incidences of infection or menstrual changes were reported in either group. In the cryotherapy group, two women developed facial flushing, dyspnea and a diffuse body rash after treatment. Owing to the rash, the above symptoms were successfully treated immediately with an intramuscular injection of dexamethasone. Two women conceived naturally after thermocoagulation and had a term delivery. Three women conceived naturally after cryotherapy: two had a term delivery, and one was in the third trimester at the time of manuscript preparation.

## Discussion

In the present study, women treated with thermocoagulation for CIN2/3 had clinical cure rates of 97.1% and 98.5% at the 4‐ and 8‐month follow‐up, respectively. These rates are similar to the pooled cure rates of approximately 95% for CIN2/3 disease treated by thermocoagulation reported in a meta‐analysis of 13 studies,^9^ and are consistent with the British Society for Colposcopy and Cervical Pathology requirements for acceptable cure rates.^12^, ^13^ At 4 and 8 months after thermocoagulation, the cytology‐negative rate was 80.8% and 100%, respectively, and the HPV‐negative rate was 74% and 87%, respectively. These proportions are also consistent with other studies.^10^, ^14^ The cytology‐negative and HPV‐negative rates increased between 4 and 8 months, which might reflect the time needed to clear both HPV infection through the autoimmune system after treatment and also coincidental vaginal HPV.^15^

In the present study, the cytology‐negative rate was higher for thermocoagulation than for cryotherapy at 8 months after treatment, but there was no difference between the groups in HPV‐negative or clinical cure rate. The therapeutic effect of the two treatment modalities for treating high‐grade cervical lesions was equivalent. The cure rate of cryotherapy for cervical lesions is reported to be comparable to that of LEEP in hospitals.^4^ Although the overall patient cost for cryotherapy will be considerably less than that for LEEP (procedure plus pathology), cryotherapy has specific problems related to the cost and availability of medical grade gases, especially in remote areas where access to gas supplies is limited and transportation difficult.

The Liger thermal coagulator (comprising a charger and device in a small briefcase) is easy to carry and inexpensive. It retains many advantages of cryotherapy such as a quick learning curve for its safe operation and a very low risk of harm. Owing to its portability, it is suitable for use in almost all regions of the world. The treatment time for thermocoagulation is also significantly shorter than that for cryotherapy. There is also no probe adhesion to cervical tissue after thermocoagulation, further adding to its ease of use. The thermal coagulator can be separated from the cervical tissue immediately, whereas the cryoprobe cannot be removed until the tip temperature is reversed, releasing it from the cervical tissue.

In cryotherapy, if the cervical lesion exceeds the area of the cryoprobe, it is laborious to retreat the lesion and the timing of retreatment is not always clear. Generally, a lesion larger than the cryoprobe is viewed as a contraindication to cryotherapy. In the present study, the diameter of the cryotherapy probe was 19 mm with a 3‐mm nipple. The probe used for the initial application of thermocoagulation was also 19 mm with a 3‐mm nipple, but a flat probe was used for additional applications (each 45 s) in cases where the cervical lesion exceeded the coverage area of the nipple probe. The present results showed that the number of treatment applications per patient for thermocoagulation did not differ relative to cytology‐negative, HPV‐negative and clinical cure rates at follow‐up. In other words, thermocoagulation was equally effective regardless of lesion size. In addition, because multiple thermocoagulation treatments were not superimposed, the treatment depth was unlikely to be excessive. In the present study, the four women referred from cryotherapy to thermocoagulation due to large lesions underwent four applications of thermoablation and no lesions were found at either the 4‐ or 8‐month follow‐up.

Singh *et al*.^11^ reported rates of complications of 1.1% (1/89) for thermocoagulation and 1.5% (1/65) for cryotherapy. In both cases, the complication was a local infection, which was treated successfully with a broad‐spectrum antibiotic. In the present study, the women reported mild lower abdominal pain during both types of treatment. The degree of pain caused by thermocoagulation was slightly higher than that caused by cryotherapy, possibly because cryotherapy can reduce nerve sensitivity. All women tolerated the pain without using anesthesia. Both thermocoagulation and cryotherapy cause local vasoconstriction of the cervix to stop bleeding; therefore, there was no vaginal bleeding during treatment in either groups. Two women developed an allergy‐like reaction (probably vasovagal reaction, retrospectively) after cryotherapy, but no such condition occurred in the thermocoagulation group. As compared with cryotherapy, thermocoagulation had a shorter duration and smaller quantity of post‐therapy discharge, but the women reported bleeding for a longer duration after treatment. Nevertheless, the amount of vaginal bleeding was small and controlled with standard menstrual products. No women developed an infection or had menstrual changes after either treatment. Regarding longer‐term follow‐up, two women conceived naturally after thermocoagulation, and three women conceived naturally after cryotherapy. Thermocoagulation and cryotherapy are reported to create minimal changes to the anatomy of the cervix, without causing adverse pregnancy outcomes such as spontaneous abortion or premature delivery.^16^

The major strength of the present clinical trial is that it was conducted in the colposcopy clinic of the Center for Cervical Diagnosis, at Peking University Shenzhen Hospital. This center, the oldest of its type in China, has much experience in conducting clinical trials carefully according to protocol. In addition, because of patient volumes and many international collaborations, the center's cervical pathology team has extensive experience.

The major limitation of the study is that the number of women with CIN3 was relatively small and the follow‐up time was short. Therefore, studies with more CIN3 cases and longer follow‐up are needed.

In summary, thermocoagulation with the Liger HTU‐100 instrument was found to be as effective and safe as cryotherapy for treating high‐grade CIN. The thermal coagulator is easier to use, requires much shorter treatment times, and is less costly because it does not require the purchase or transport of medical treatment gases.

## Disclosure

None declared.

## Author contributions

L. D. contributed to literature search and review; data extraction, interpretation and analysis; and manuscript preparation. H. D., J. L. B. and X. Q. contributed to study design and initiation. Z. L., A. X., S. L. and L. Z. contributed to patient treatment and follow‐up. C. W. participated in pathologic diagnosis. R. W. contributed to study initiation and design; and manuscript revision. All authors reviewed and approved the final version of the manuscript.
